# Reproductive Physiology and Reproductive Parameters of Male Owston’s Civets (*Chrotogale owstoni*)

**DOI:** 10.3390/ani16172677

**Published:** 2026-08-26

**Authors:** Veronica B. Cowl, Isabel Callealta, Christa S. van Wessem, Tullis Matson, Zak Showell, Susan L. Walker, Rebecca Mogey, Kathy Baker, Scott Bird, Jane Hopper, Katie L. Edwards

**Affiliations:** 1 North of England Zoological Society (Chester Zoo), Caughall Road, Upton-by-Chester, Chester CH2 1LH, UK; 2 ECOlifes, 11140 Cadiz, Spain; 3 Bay Vet Group, 1 Yannons Ct, Yannons Rd., Paignton TQ4 7HU, UK; 4 Nature’s SAFE, Ash Lane, Whitchurch, Shropshire SY13 4BP, UK; 5 Shaldon Wildlife Trust, Ness Dr, Shaldon, Teignmouth TQ14 0HP, UK; 6 Newquay Zoo, Trenance Ave, Newquay TR7 2LZ, UK; 7 Thrigby Hall Wildlife Gardens, Filby Rd., Thrigby, Great Yarmouth NR29 3DR, UK; 8 Port Lympne Hotel & Reserve, Aldington Rd., Lympne, Hythe CT21 4LR, UK

**Keywords:** fertility, Owston’s civet, reproduction, reproductive organ measurements, semen parameters, testosterone, wildlife endocrinology

## Abstract

Ex situ breeding has been identified as a key action for conserving the Endangered Owston’s civet (*Chrotogale owstoni*). Historically, however, the species has had poor reproductive rates under managed care. In males, it is necessary to understand testicular function to identify factors that affect reproductive success. Here, we conducted long-term faecal testosterone (Tt) monitoring, ultrasonography of the reproductive tract, and sperm assessments in five Owston’s civets held in zoos in the United Kingdom to gain insight into male fertility. Male Tt metabolite concentrations were higher in the winter and spring months, with significant cyclical increases in Tt from December to January, corresponding to the start of the in situ breeding season. No significant reproductive pathologies were identified among males, with the exception of mild irregular margins and hyperechoic foci in the prostate of one male. Semen was collected through urethral catheterization and electroejaculation from three out of four males, with repeat samples collected from one male. Urethral catheterization yielded small but highly concentrated samples, and is a simple, cost-effective, and field-friendly method of sperm collection in the species. We highlight the utility of reproductive monitoring to gain insight into male fertility.

## Simple Summary

A key action for the conservation of the Endangered Owston’s civet (*Chrotogale owstoni*) is the development of a healthy and robust population managed under human care. Historically, this species has not bred well in zoos; understanding the reproductive biology of the species is essential to improve reproduction and to develop assisted reproductive technologies. We monitored faecal testosterone (Tt) in five male civets and conducted reproductive examinations on four males, including three repeat examinations on one male. We collected sperm and male reproductive organ measurements to gain a better understanding of how Tt, sperm, and reproductive organs relate to fertility. Faecal Tt concentrations were higher over the winter and spring months, corresponding to the time when the species typically breeds in its home range. We collected viable sperm from most of the males and did not identify any significant reproductive issues that would limit reproduction in this population.

## 1. Introduction

Zoos are increasingly being recognised for their contributions to endangered species conservation, notably through their expertise in the care and management of small and fragmented populations [1]. As species propagation is a focus for ex situ conservation [2], an understanding of reproductive biology is essential to identify which factors contribute to reproductive success and which individuals are most likely to breed [3,4]. A basic knowledge of reproduction is also required to develop assisted reproductive technologies (ARTs), such as artificial insemination or in vitro fertilisation, which have the capacity to increase reproductive rates in subfertile populations and can connect in situ and ex situ populations through the preservation and movement of gametes rather than individuals [3,4]. However, many wildlife species are relatively understudied and, as significant differences in reproductive biology can occur in species of the same family or genus, intensive research is required on each unique species [3]. To better optimise natural and assisted breeding in males, a comprehensive understanding of reproductive physiology, reproductive tract anatomy, and semen characteristics are essential.

Testosterone (Tt) is the primary male reproductive hormone, regulating sexual maturity, reproductive behaviour, spermatogenesis, and reproductive success [5,6,7,8]. Hormones have traditionally been measured in blood; however, it is often unfeasible to collect regular, longitudinal samples from animals managed in zoos. As such, non-invasive hormone monitoring, for example by monitoring hormones in faeces, has become more popular in wildlife reproduction [9]. Faecal Tt has been measured in a range of mammalian species including carnivores [10,11,12], primates [5,7], ungulates [13,14], and elephants [15]. In addition to non-invasive hormone monitoring, reproductive assessments that include ultrasonography and semen assessments further provide additional information on the morphology and pathology of the reproductive tract and individual fertility [4,16]. As most species ex situ are not trained for human handling, ultrasonography and reproductive assessments are typically conducted under anaesthesia and are therefore considered minimally invasive.

The Owston’s civet (*Chrotogale owstoni*) is a small nocturnal Asiatic viverrid, and the only member of its genus [17]. Classified by the International Union for the Conservation of Nature (IUCN) Red List as Endangered in 2016 [18], its in situ range is restricted to Vietnam, Lao PDR, and southern China, where it faces severe threats of indiscriminate hunting and has no functionally protected land [17]. A significant decline of the in situ population occurred between 2006 and 2013 [18], which has led to the Owston’s civet being considered ‘one of the most threatened carnivore species in the world’ [17]. In 2019, stakeholders developed an IUCN Conservation Strategy for Owston’s civets, where establishing a healthy ex situ population for the species was identified as a priority, with the specific objective of understanding factors that contribute to reproductive success [19]. A dedicated ex situ conservation breeding programme for Owston’s civets was established in Vietnam in 1995, and in 2005, three pairs were sent to the United Kingdom (UK) [20]. Currently, the managed ex situ Owston’s civet population consists of four animals in the UK (M, F: 2, 2), and, owing to a recent population boom, of 47 animals at Save Vietnam’s Wildlife, Vietnam (M, F, U: 16, 15, 16) [21].

The most studied Asiatic civet species is the Least Concern common palm civet (*Paradoxurus hermaphroditus*), where male and female reproductive physiology [22,23], macroanatomy of male reproductive tract [24], and sperm measurements have been reported [25,26]. In comparison, there is a paucity of knowledge on the life history and reproductive biology of the Owston’s civet owing to the elusive nature of the species. Owston’s civets are considered seasonal breeders, primarily breeding between January and March, with most births between April and July [19,21]. However, some reports extend the breeding season to November [27]. Ex situ, the reproductive lifespan of male Owston’s civets spans from 1.8 to 15.1 years [21]. To date, no information on the reproductive physiology or reproductive parameters of Owston’s civets have been published. Here, we present the results of over seven years of reproductive monitoring in five male Owston’s civets in the UK, including long-term faecal hormone monitoring, reproductive tract ultrasonography, and semen assessments.

## 2. Materials and Methods

### 2.1. Animals

This study was approved by the Chester Zoo Research Committee (CZ_00517 approved 1 March 2019) as well as the respective institutions involved in this study. Male captive born Owston’s civets (N = 5) were housed at four European Association of Zoos and Aquaria zoos: Newquay Zoo (NZ), Port Lympne Wild Animal Park (PL), Shaldon Wildlife Trust (SWT), and Thrigby Hall Wildlife Gardens (TH; all UK). Males were between 1.60 and 13.92 years old at the start of sample collection and included proven and non-proven males, who were generally paired with parous and/or nulliparous females during the breeding season (Table 1).

### 2.2. Faecal Tt Metabolite Concentrations

Each institution aimed to collect at least one sample per week for each male. A total of 801 faecal samples were collected from the males in the study, ranging between 29 and 291 samples for each male. Where necessary, institutions added faecal markers to the civet diet, such as glitter or lentils, to identify the individual the sample came from [28]. Each sample was placed in a sample bag, labelled with individual ID and the date of collection, and frozen at −20 °C until they were ready to be shipped to the Chester Zoo Conservation Physiology Laboratory (UK) for analysis. We immediately placed samples in a freezer (−20 °C) upon receipt to the lab.

We lyophilized and extracted faecal samples using methods adapted from Hunt and Wasser [29] and Scarlata et al. [30]. Briefly, we crushed and sifted lyophilised faecal samples using 500 µm fine mesh sieves before storing them in labelled tubes at −20 °C. To extract each sample, we weighed 0.10 g (0.095–0.150 g) of dry faecal powder into a glass boiling tube before adding 5 mL of 90% methanol solution. We vortexed the samples prior to shaking them overnight on a multi-tube vortex at 1000 RPM. Next, we centrifuged samples at 1500× *g* for 20 min before pouring off the supernatant to be evaporated under air in a 50 °C water bath until dry. Once dry, we added 1 mL of 100% methanol to each boiling tube, vortexed, and sonicated each sample for 15 min. We poured off extracts into labelled and sealed plastic tubes, where they were kept frozen until further analysis.

We assessed faecal Tt metabolite concentrations using double-antibody enzyme immunoassays (EIAs) incorporating plates pre-coated with 0.01 mg/mL secondary goat-anti rabbit immunoglobulin antibody (A009, Arbor Assays, Ann Arbor, MI, USA) and polyclonal rabbit antibodies against Tt (R156/7, C. Munro, University of California, Davis, CA, USA) adapted from Munro and Stabenfeldt [31]. We diluted standards (50 μL, 2.34–600 pg/mL, Sigma-Aldrich, Gillingham, UK), controls and samples (1:10–1:1000, as required) in phosphate buffer (X065, Arbor Assays) and added Tt horseradish peroxidase (50 μL, 1:200,000, C.J. Munro, University of California, Davis, CA, USA) to all wells. Next, we added the primary anti-Tt antibody (50 μL; R156/7 1:600,000) to all wells except for the non-specific binding wells prior to incubation in the dark for 2 h at RT while shaking at 400 RPM. We washed plates five times with wash buffer (X007, Arbor Assays) to remove unbound components before adding chromogen solution containing TMB (3,3′,5,5′-tetramethylbenzidine; 100 μL, X019, Arbor Assays) to each well. We incubated plates for 30 min at RT and halted the reaction by addition of stop solution (50 μL; X020 Arbor Assays). We determined optical densities at 450 nm with a reference of 630 nm. Cross reactivities are reported by de Catanzaro et al. [32].

We biochemically validated assays through parallelism and matrix interference assessments. Serial dilutions of faecal extract yielded a displacement curve parallel to the standard curve (y = 1.054x − 2.02, R^2^ = 0.994, F_1,7_ = 1178.502, *p* < 0.0001; Figure S1). There was no evidence for matrix interference; spiking assay standards with a known amount of diluted faecal extract to synthetic standards did not alter the amount observed (y = 1.15x ± 8.49, R^2^ = 0.97, F_1,7_ = 246.40, *p* < 0.0001). The inter-assay coefficients of variation (CVs) for high and low synthetic, and biological controls were 8.38%, 11.97%, and 11.84%, respectively. All samples, standards, and controls were run in duplicate, and CVs were maintained below 10%.

### 2.3. Reproductive Assessments

In 2022, we conducted reproductive assessments during the breeding season in males 1–4. Male 2 received two additional reproductive assessments in the 2024 breeding season during attempts at artificial insemination. Males 1 and 4 were immobilised with a combination of medetomidine (Domitor 1 mg/mL, Vetoquinol, Towcester, UK; 0.05 mg/kg) and ketamine (Ketavet 100 mg/mL, Zoetis, Leatherhead, UK; 2 mg/kg; Table S1). To improve anaesthetic and sperm retrieval outcomes, subsequent individuals (males 2 & 3) were immobilised in a chamber with sevoflurane and maintained with gas and a supplemental intramuscular dose of 0.03 mg/kg medetomidine [33,34] (Table S1). All individuals were intubated (size 3.5–4.5 mm endotracheal tubes) and maintained on gaseous anaesthetic (Table S1). All animals were given 0.1 mg/kg meloxicam (Metacam 5 mg/mL, Boehringer Ingelheim, Bracknell, UK) subcutaneously during the procedure. At the end of each procedure, the animal was recovered in its carrier and reversed with atipamezole (Atipam 5 mg/mL, Dechra, Shrewsbury, UK) at five times the dose of medetomidine given (Table S1).

We subjectively calculated body condition score (BCS) based on body fat, where individuals were assigned a score of 1 to 9 (1 being emaciated, 5 being ideal, and 9 being obese; Table S2) [21]. Reproductive assessments consisted of a macroscopic evaluation of the penis and testes, percutaneous ultrasonographic examination (Versana Active, General Electric Healthcare (Düsseldorf, Germany), 4–13 MHz linear probe [12L-RS]), and microscopic semen assessment. In all animals, we examined the testes, epididymis, and prostate. We recorded short video sequences of 6–10 s onto the ultrasound drive during the examinations which were retrospectively used to measure organ area (length × width) or single diameters (epididymis) of both sides. To establish the health status of each animal we also conducted full clinical health assessments during each procedure which included routine full biochemistry and haematology panels and ultrasonography of the urinary bladder, kidneys, adrenal glands, spleen, liver, pancreas, stomach and the small and large bowel where possible.

We initially collected semen through urethral catheterization (UC) [33,34], by inserting a sterile commercial 1.3 × 150 mm cat urinary catheter (Buster, Kruuse, Langeskov, Denmark) in the urethra. After performing a rectal enema, we collected additional semen samples through electro-ejaculation (EE); we applied three sets of 10 electrical pulses (2 Volts), transrectally over the prostate and along the urethra, using a portable battery-driven system (El Toro 2, Electronic Research Group, Johannesburg, South Africa) and a 220 mm long and 9.5 mm diameter domestic dog and cat transrectal probe (Minitube, Tiefenbach, Germany). We collected semen samples in pre-warmed 1.5 mL capped Eppendorf vials and assessed samples for total volume, sperm concentration, progressive motility, viability, and morphology. In 2022, we pooled UC and EE samples, and in 2024, we assessed samples collected via UC and EE separately for volume and concentration. We calculated sample volume using a pipette. We assessed sperm viability and morphology by preparing a smear by mixing 1:2:1 parts eosin:nigrosin:sperm [35] and evaluated at least 100 sperm under phase-contrast optics. Sperm were characterised as normal or as having primary or secondary defects as per Zambelli and Levy [36]. We assessed all other sperm parameters using a mobile computer-assisted semen analyser (iSperm mCASA^®^, Aidmics Biotechnology Co., Ltd., Taipei City, Taiwan). Briefly, we diluted samples in 20–50 µL of prewarmed 37 °C medium (in 2022: Tris and egg-based extender with 6% glycerol, Elite Kennel Fertility freezing medium, UK; in 2024: cell culture medium, Medium 199, Sigma-Aldrich, Darmstadt, Germany) before loading a small aliquot (7.5 µL) on to the iSperm chip for analysis. In domestic species, sperm is usually classified as normal when progressive motility and normal morphology are >70–80% and vitality is >60% [36]. To the authors’ knowledge, to date there are no established normal parameters for civets.

### 2.4. Statistical Analyses

We conducted all analyses using R (version 4.6.0) [37]. We used linear mixed models (LMMs) in the base *stats* package (version 4.6.0) to compare Tt metabolite concentrations across male age, reproductive status, and meteorological season (spring: March–May, summer: June–August, autumn: September–November, winter: December–February). Male 1 was considered an older male, males 2, 3, and 5 were considered adults, and male 4 was considered a young adult. In all models, individual ID was the random factor. Male Tt was log-transformed to fit assumptions of normality. We assessed model fit by looking at QQ plots, the normality of residuals, and by comparing fitted versus residual values. We calculated the conditional R^2^ for each model using *MuMIn* package (version 1.48.19) to assess model fit [38]. We used a stepwise selection process to build models, only including significant variables (*p* < 0.05) from univariate models in subsequent multivariate models. Each model was ranked using the Akaike’s information criterion (AIC) whereby the model with the lowest AIC is the most optimal. We conducted Tukey post hoc tests only where main variables were significant, using the package *multcomp* (version 1.4-30) [39]. To assess the relative importance of differences between individuals in the study, we calculated the adjusted intraclass correlation coefficient (ICC; random effect variance divided by the residual variance) using the *performance* package (version 0.17.0) [40].

We used changepoint analysis to identify significant changes in the mean and variance of Tt metabolite concentrations over time in males with continuous data over 12 months (males 2 and 5) using the *changepoint* package (version 2.3) [41]. We used an iterative process to detect changepoints (Pruned Exact Linear Time (PELT) algorithm) which assesses the cost of adding a changepoint at every position, keeping only changepoints with significant changes to the mean and variance. As there is no limit to the number of changepoints identified using PELT, we used a BIC penalty of 22 and searched for a minimum segment length of 15 to ensure that only significant changes in segments were identified. We paired the PELT algorithm with a changepoint for a range of penalties (CROPS) algorithm [42], which reruns the PELT algorithm at a range of penalties (5 to 50). As the addition of new changepoints is penalised, the CROPS algorithm identifies the point at which the addition of new changepoints is no longer computationally efficient. Where slightly different changepoint locations were identified using each method, we selected the intermediate location as a changepoint location. If additional changepoints were identified using CROPS, we reran the CROPS algorithm at a range of penalties (ranging from 10 to 100) and compared the CROPS elbow plot to identify the optimum number of changepoints in the data set. We classified segments as having high or low Tt metabolite concentrations; we only include full segments in mean calculations of the duration and Tt metabolite concentration of each segment. Lastly, we used a generalised least squares (GLS) model using the package *nlme* (version 3.1-169) to identify which segments differed significantly from one another [43]. Each individual was evaluated separately. Male Tt was log-transformed to fit assumptions of normality.

We used descriptive results to summarise the findings of the reproductive assessments. Where multiple measurements were taken from the same male, we used mean values for all subsequent analyses. Owing to small sample sizes, we compared the area of reproductive organ area and semen concentration across males using Mann–Whitney-U tests (reproductive status) and Spearman’s correlation tests (age, BCS, Tt metabolite concentration). Across our results, we used the mean ± standard error to summarise inter-individual differences and the mean ± standard deviation (SD) to describe intra-individual differences. Generative AI was used for the purpose of writing part of the code used to calculate the ICC, for changepoint analysis, to develop the figure illustrating LMM results, and for reference formatting (Claude, Anthropic, San Francisco, CA, USA, Sonnet 4.6, 2026).

## 3. Results

### 3.1. Faecal Tt Metabolite Concentrations

**Figure 1:**
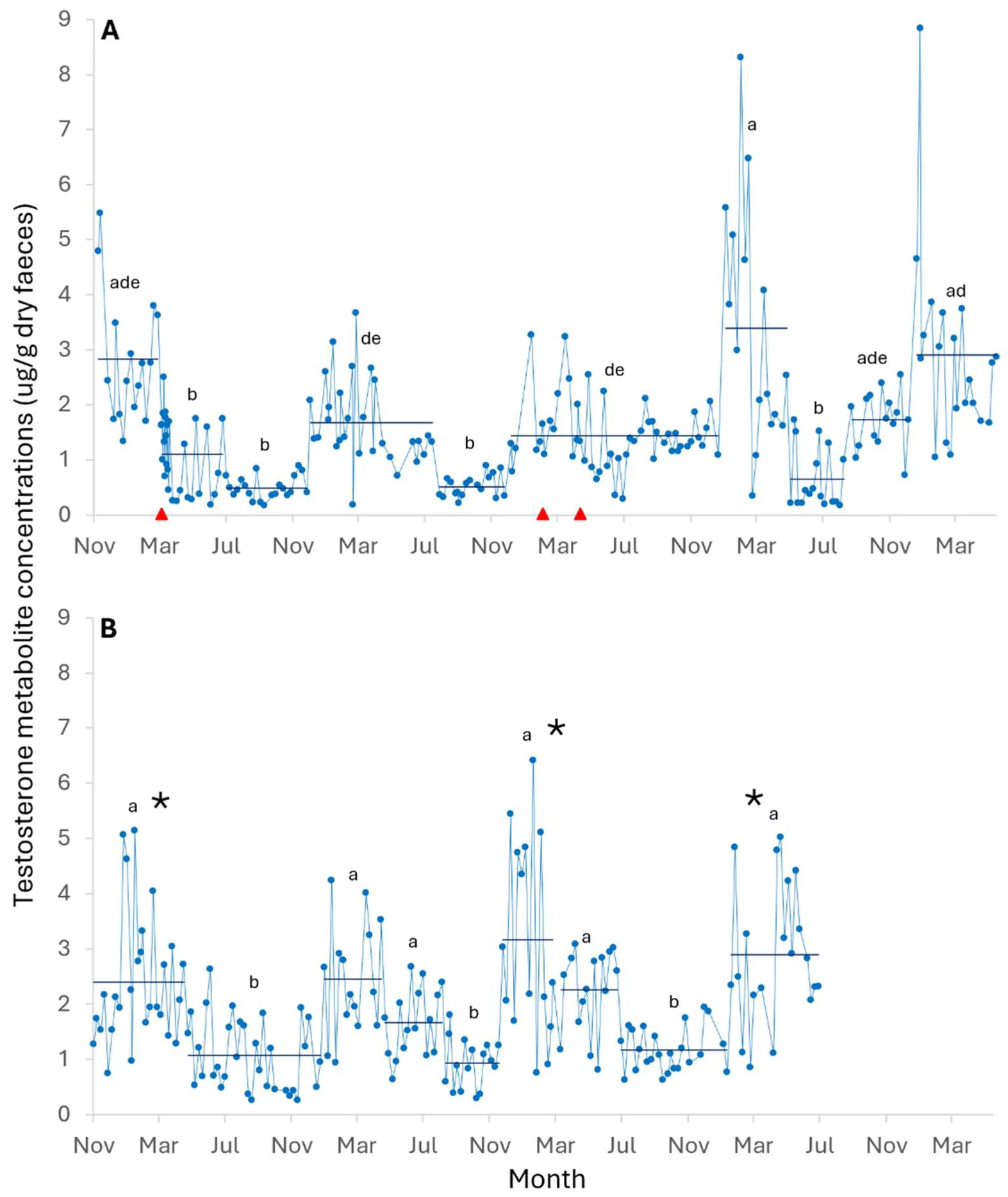
Weekly faecal Tt metabolite concentrations across multiple years for (**A**) non-proven male 2, and (**B**) proven male 5. Horizontal lines highlight mean Tt metabolite concentrations for that segment and denote significant changes in the mean and variance, red triangles denote dates of reproductive assessments, and black asterisks denote conceptive breeding seasons. Letters above segments identify segments that are statistically the same (*p* > 0.05).

Faecal Tt metabolite profiles for males 2 and 5 highlighted several cyclical changes to the mean and variance of Tt metabolite concentrations throughout the year (Figure 1). Generally, Tt metabolite concentrations increased significantly between December and January, before decreasing over the summer and autumn months. Interestingly, changepoint analysis identified intermediate shifts in mean Tt concentration either preceding or following high Tt metabolite concentrations in both profiles (Figure 1). These intermediate periods generally did not differ significantly from higher Tt metabolite concentrations. In the non-proven male, Tt metabolite concentrations generally increased and decreased earlier in the year compared to the proven male. In the proven male, Tt metabolite concentrations increased earlier in conceptive years compared to the only non-conceptive year. In the non-proven male, Tt metabolite concentrations were elevated for 205.25 ± 128.63 days, with concentrations of 1.82 ± 1.19 ug/g dry faeces, and low for 119.25 ± 21.06 days, with mean concentrations of 0.73 ± 0.54 ug/g dry faeces (Figure 1). In the proven male, elevations of Tt metabolite concentrations were shorter (101.5 ± 6.35 days) but reached higher concentrations (2.37 ± 1.24 ug/g dry faeces) than the non-proven male. Periods of low Tt metabolite concentrations were longer (179.00 ± 74.30 days) and had higher Tt metabolite concentrations (1.07 ± 0.53 ug/g dry faeces) in the proven male compared to the non-proven male. Individual profiles for males 1, 3, and 4 are provided in the Supplementary Materials.

**Figure 2:**
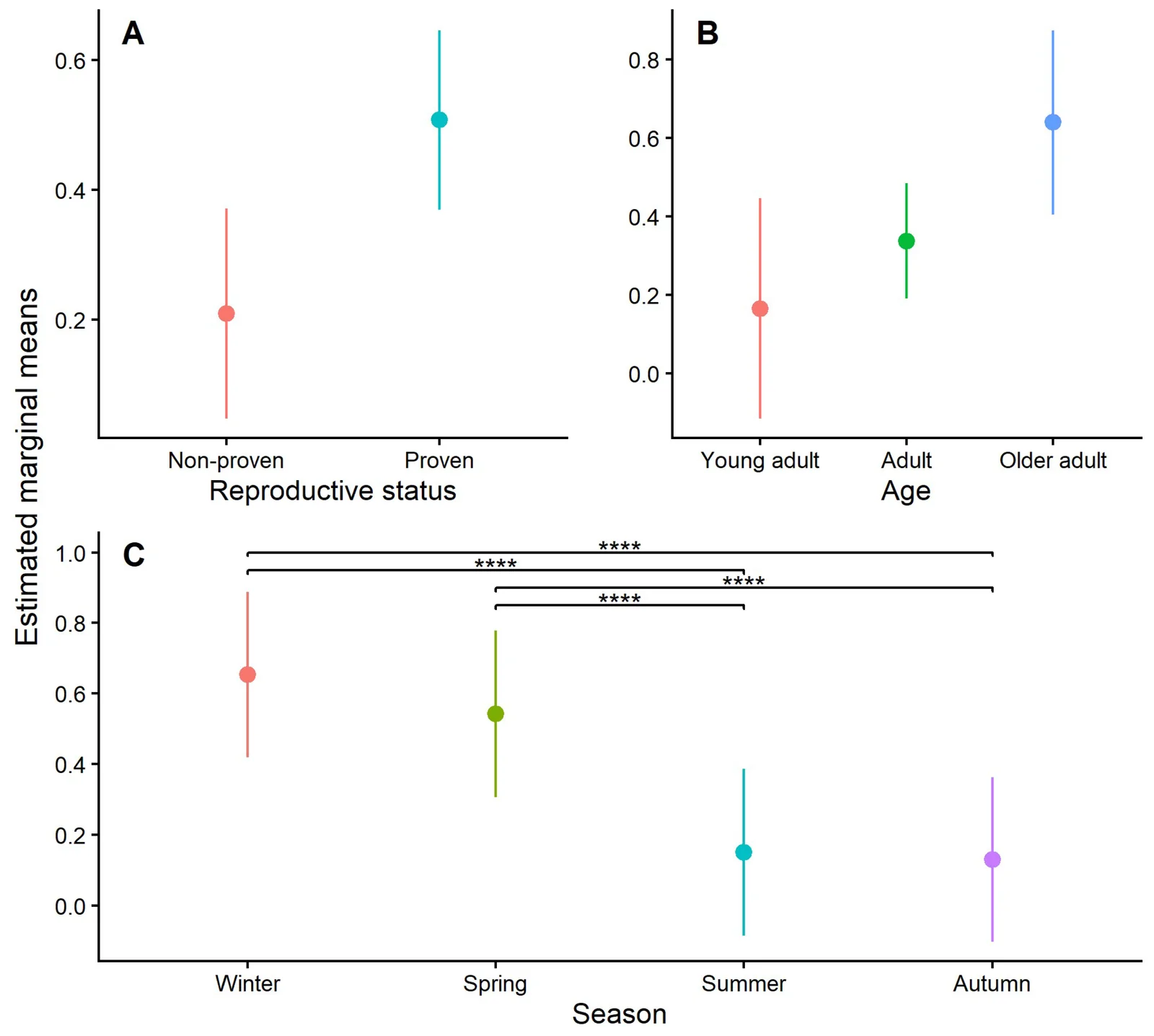
Estimated marginal means from linear mixed effects models with 95% confidence intervals of Tt metabolite concentrations across (**A**) reproductive status, (**B**) age, and (**C**) season. Significance is denoted with bars across boxplots (*p* < 0.0001 = ****, NS = not illustrated).

We found a significant effect of season on male Tt metabolites, where concentrations were significantly higher during winter and spring than in summer or autumn (Figure 2, Table 2). While proven and older males had slightly higher Tt metabolite concentrations, neither factor was significant (*p* > 0.05; Table 2). Each model had a low R^2^ and did not improve the AIC compared to the null model; as such, neither reproductive status nor age have a high explanatory power for our dataset. However, the low intraclass correlation coefficient, combined with the high standard error of the random variance intercept suggest that significant variation occurs within individuals rather than between individuals (Table 2).

### 3.2. Reproductive Assessments

#### 3.2.1. Macroscopic Evaluation and Ultrasonography of the Gonads and Accessory Glands

**Figure 3:**
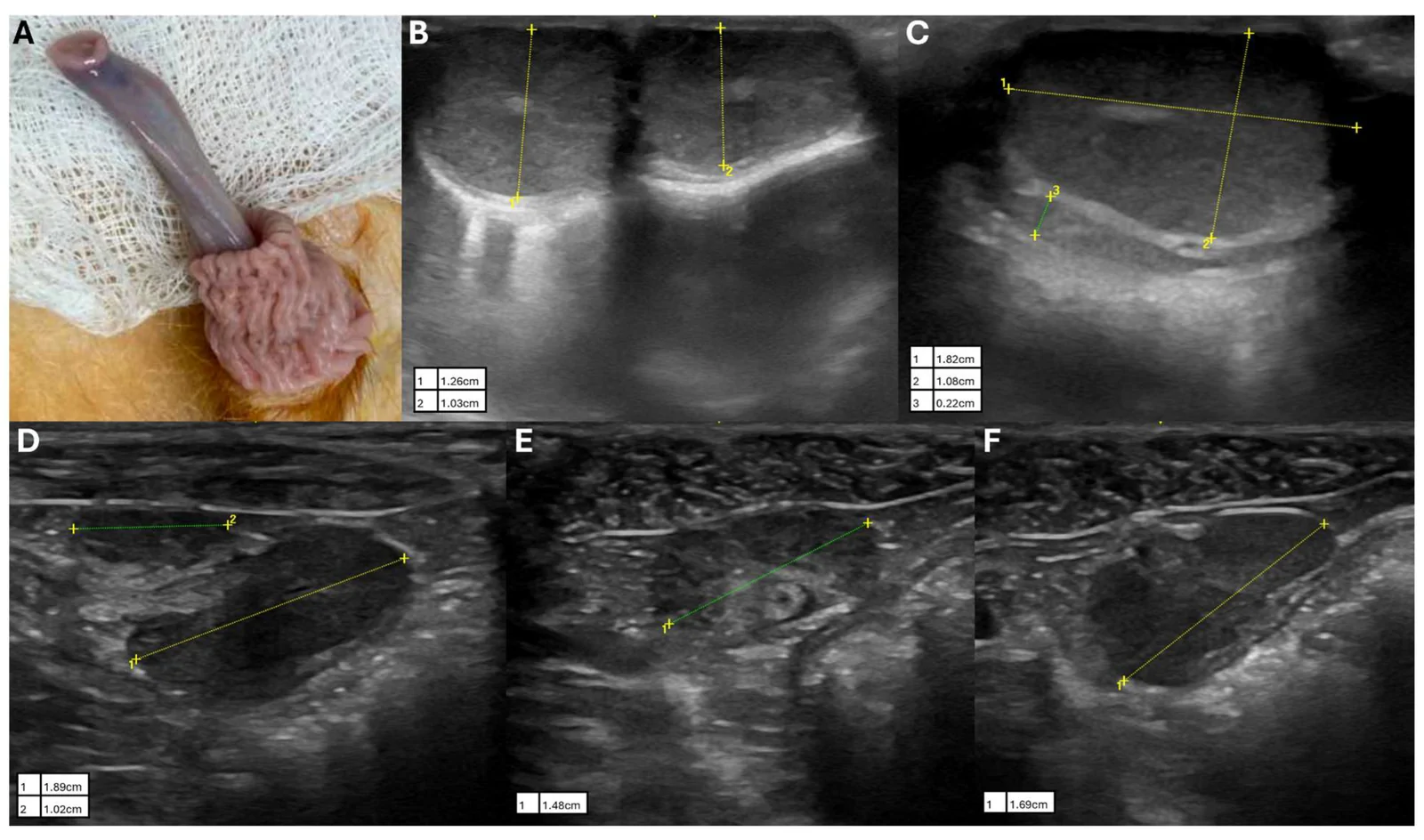
Macroscopic image of the (**A**) penis protruding out of the prepuce and sonograms of the (**B**) left and right testis (transversal view), (**C**) one testis and the epididymis (longitudinal view), (**D**) cranial and caudal prostatic lobes (longitudinal view), (**E**) cranial prostatic lobe (transversal view), and (**F**) caudal prostatic lobe (transversal view) of male 2. Yellow and green lines indicate length/width of the reproductive organ in cm. Numbers in tables correspond to the numbers within each image.

All males had two descended testicles, and no abnormalities were noted on palpation. Upon macroscopic evaluation, no male was affected by phimosis, and no lacerations or adhesions were detected on the penis, which does not have a baculum in this species. With the exception of male 3, no significant abnormalities were noted on the ultrasound scans performed in this study. The testes and epididymides of each male were normal in shape and echogenicity. No major pathologies were noted and there were no notable size differences between testes of each male. The prostate in this species seems to be multilobulated with one cranial lobe located ventral to the urethra and a larger, caudal lobe located dorsal to the urethra. In males 1, 2, and 4 the prostate was hypoechoic, and uniform in echotexture, with smooth margins, and did not show any cysts or other clinically significant abnormalities (Figure 3). In male 3, the prostate had mild irregular margins and a couple of hyperechoic foci suggestive of mild chronic pathology. The mean testis area across both testes and all males was 3.05 ± 0.06 cm^2^ (Table 3). Testis area, prostate area, and epididymis width did not appear to vary with age (testis: Spearman’s ρ = −0.80, *n* = 4, *p* = 0.33; prostate: Spearman’s ρ = 0.21, *n* = 4, *p* = 0.78; epididymis: Spearman’s ρ = −0.32, *n* = 4, *p* = 0.68), BCS (testis: Spearman’s ρ = 0.32, *n* = 4, *p* = 0.68; prostate: Spearman’s ρ = 0.33, *n* = 4, *p* = 0.67; epididymis: Spearman’s ρ = 0.50, *n* = 4, *p* = 0.50), Tt metabolite concentration (testis: Spearman’s ρ = −0.50, *n* = 3, *p* = 1.00; prostate: Spearman’s ρ = −0.50, *n* = 3, *p* = 1.00; epididymis: Spearman’s ρ = 0.87, *n* = 3, *p* = 0.33), or reproductive status (testis: W = 3, *p* = 0.67; prostate: W = 2.5, *p* = 1.00; epididymis: W = 2, *p* = 1.00) on the date of measurement. However, given the small sample size, statistical power is limited.

#### 3.2.2. Semen Parameters

We collected a total of six samples from males 1–4, including three repeat semen collections on separate occasions in male 2 (Table 3 and Table 4). No spermatozoa were seen in the samples collected from male 3. Mean sample volume across all males was 37.69 ± 9.88 µL. There was a low proportion of normal sperm across samples (<10–20%), with the exception of the second reproductive assessment in male 2, where 47% of sperm were normal. Primary and secondary defects across samples primarily consisted of proximal and distal droplets (88–97% of abnormalities across males). There was no significant relationship between semen concentration and age (Spearman’s ρ = −0.60, *n* = 4, *p* = 0.33), BCS (Spearman’s ρ = 0.60, *n* = 4, *p* = 0.37), Tt metabolite concentration (Spearman’s ρ = −1.00, *n* = 3, *p* = 0.33), or reproductive status (W = 4, *p* = 0.33) in the pooled samples. Again, given the small sample size, statistical power is limited. Although samples collected via UC were smaller than those collected via EE, they were more highly concentrated (Table 4).

## 4. Discussion

Understanding the reproductive biology of a species is fundamental to advancing the reproductive potential of ex situ populations [3]. Here we present findings of long-term reproductive monitoring and reproductive assessments in the Endangered Owston’s civet. We identified clear, circannual Tt cycles in male Owston’s civets, with increases in Tt metabolite concentrations coinciding with the start of the January breeding season. Viable sperm was collected from most of the males through both urethral catheterisation and electroejaculation. Furthermore, we provide male ultrasonographic reproductive organ measurements and semen parameters in proven and non-proven Owston’s civet males.

The males in this study had defined, circannual Tt peaks between December and May, coinciding with the primary reported breeding season in free-ranging individuals [19]. Circannual Tt cycles are observed in other seasonally breeding male carnivores including giant pandas (*Ailuropoda melanoleuca*) [12,44], black bears (*Ursus americanus*) [45], red pandas (*Ailurus fulgens fulgens*) [10], and the common palm civet [22], where Tt metabolite concentrations increase during the dry season between December and April. In many seasonally breeding species, Tt production is tightly regulated by photoperiod [12,46,47], climate [22], and food availability [48] so that breeding coincides with female periods of receptivity [12] and resource availability [22], improving reproductive success and offspring survival [12,47]. In this study, the species’ breeding season appears to be conserved in animals housed in the UK, suggestive of a strong endogenous circannual rhythm given the difference in climate [49], photoperiod [50], and food availability [51] between the UK and the species’ in situ range.

Our results highlight that intra-individual differences account for more of the variation in Tt metabolite concentrations than age or previous breeding success. Although we did not investigate the association between social housing and testosterone, in free-ranging giant panda, male Tt concentrations during the breeding season are associated with female receptivity and male competition [12]. Similarly, in southern white rhino (*Ceratotherium simum simum*), access to receptive females, but not previous reproductive success, is associated with increased Tt metabolite concentrations [13]. It is conceivable that females may not have been receptive, or a lack of male competition may have contributed to a poor reproductive success in the UK ex situ population. Interestingly, Tt metabolite concentrations in male 2 did not show the same cyclical changes during the third year of hormone monitoring, during which two ovarian stimulation procedures occurred in the female. Although we cannot directly infer a causal relationship, we speculate that these procedures may have affected species typical reproductive cues. During this study, no significant health issues were identified in male 2; in other mammals, however, the maintenance of consistently high Tt concentrations is energetically costly and can lead to increased risk of injury, loss of fat stores, compromised immune system function, and oncogenic effects [8]. As possible costs of elevated Tt concentrations have not been directly studied in this species, individual male Tt and health should be carefully monitored in future to ascertain whether consistently elevated Tt concentrations occur more frequently and whether any resulting negative health effects have been observed.

We did not find a significant relationship between reproductive organ measurements, semen parameters, reproductive status, age, BCS, or Tt metabolite concentrations. Given our limited sample size, this is not particularly surprising. Although not a statistically significant finding, non-proven males had slightly lower Tt concentrations and we found an increasing trend in Tt concentration with age. This contrasts to findings in common palm civets, where Tt concentrations were highest in 2–3 year-old males, declining with age, likely due to a reduction in testicular function [22]. Ex situ, Owston’s civets have been reported to sire offspring between the ages of 2 to 15 [21]. Here, we collected viable sperm from males up to almost 17 years of age, which may possibly extend the reproductive lifespan of the species. Reproductive capacity is, however, not solely related to spermatogenesis and can be affected by other behavioural and physical factors [4]. For example, nonproven males in this study had a slightly higher BCS than proven males, and anecdotally, animals in the UK are slightly heavier than male Owston’s civets in Vietnam [52]. While our sample size limits our ability to directly assess the role of body condition on fertility, high body fat is known to affect Tt production, spermatogenesis, and sperm quality in humans, ultimately affecting both natural and assisted reproduction [53,54,55]. In African lions (*Panthera leo*), while slight overconditioning did not appear to affect sperm parameters, sperm quality and quantity decreased, and the number of sperm defects increased in severely obese individuals [4]. Unfortunately, there is limited comparative literature on the effect of body condition on fertility in male wildlife species.

To date there is no published information on normal semen parameters in Owston’s civets. Here, we found a high proportion of abnormal sperm, which primarily consisted of proximal and distal droplets. Cytoplasmic droplets are frequently observed in samples collected by electroejaculation and/or from young individuals. When present in a high proportion, they are usually suggestive of incomplete epidydimal maturation [56]. In addition to a low proportion of normal sperm, the viability of samples collected during the initial reproductive assessments was low. Although we only assessed the viability of one sample during the second reproductive assessments, a much higher proportion of sperm were still viable upon assessment. Discrepancies in viability may be a result of the extender used; the first was developed for canids and contained glycerol, while the second was a cell culture medium without glycerol. Although glycerol is often used as a cryoprotectant, it can be toxic to sperm, with marked differences in sperm sensitivity to glycerol across species [57,58,59,60]. Future research into ARTs in Owston’s civets should assess the effects of different extenders on sperm survival.

Urethral catheterisation was a highly effective sperm collection method in Owston’s civets and provided a small but highly concentrated sample. Although our study has a low sample size, samples collected via UC were more than 16 times more highly concentrated than samples collected via EE, similar to findings in wild and domestic felids [33,34]. Sperm collection via UC is simple, inexpensive, and can be conducted without specialist equipment in most veterinary practices or under field conditions [34]. Samples collected through UC typically have less seminal plasma than samples collected through EE, which in other species may potentially improve cryopreservation outcomes [34,61,62]. A challenge, however, is ensuring that the catheter does not reach the urinary bladder, which can result in urine contamination and thus degradation of the sample. This can be avoided by determining the depth of the prostate ultrasonographically and by not inserting the catheter further than a predetermined depth [34]. Although no sperm was collected from male 3, he had sired offspring two years before the reproductive assessment. Copulation or spontaneous ejaculation before sample collection, amongst other causes, may have resulted in a lack of sperm in the sample.

Here, we only collected reproductive organ measurements and sperm from males during the breeding season, and we are therefore unable to determine how testicular function and spermatogenesis vary seasonally in this species. In other seasonally breeding species, the testes undergo circannual changes in terms of function and morphology, increasing in size and spermatogenesis during the breeding season [47,63]. In some seasonally breeding species, spermatogenesis may still occur outside of the breeding season despite reduced Tt concentrations, albeit at a reduced capacity [63,64]. Further measurements from male Owston’s civets at other times of year, as well as from the Vietnamese ex situ and free-ranging populations would be useful to better identify any seasonal trends in male reproductive activity and to better define normal reproductive parameters. If spermatogenesis continues outside of the breeding season in the Owston’s civet, this may significantly increase the times of year during which semen may be preserved. Post-mortem evaluations of testicular function, as well as opportunistic semen collections may be useful in further understanding reproductive function in this species [63].

## 5. Conclusions

We found clear circannual patterns in faecal Tt metabolite concentrations in male Owston’s civets, coinciding with the main reported breeding season in and ex situ. Urethral catheterisation was a simple and effective method of semen collection in the species, yielding a highly concentrated sample. We found no obvious male-associated factors that could contribute to a lack of reproductive success in these animals. Natural breeding with the males in this study still appears to be a viable method of reproduction, though population-level success may depend on additional factors. Future research should focus on how female fertility and general animal management may affect reproduction. All individuals in this study were held in the UK; data from managed and free-ranging in situ individuals will provide valuable information on normal reproductive parameters in this species. In collaboration with the co-authors, faecal sample collection and opportunistic reproductive assessments are being conducted on the animals at Save Vietnam’s Wildlife, which will provide important information about reproductive parameters in this species.

## Figures and Tables

**Table 1:** Summary of Owston’s civet males involved in this study. Males are all captive born either in the UK or in Vietnam. Institution indicates the location where faecal samples were collected; additional animal moves may have occurred but are not reported in the table as faecal samples were not collected during that time. Y_ indicates the year of sample collection for that individual. Institutional acronyms: NZ: Newquay Zoo, SWT: Shaldon Wildlife Trust, PL: Port Lympne Wild Animal Park. Please note that we did not conduct a reproductive assessment on male 5.

			Faecal Sampling			Reproductive Assessment		
Male	Institution	Reproductive Status (Year of Offspring Birth)	Age at Start of Sample Collection (Years)	Sample Collection Duration (Years)	N Samples	Year	Weight (kg)	Body Condition Score
1	NZ	Proven (2014, 2015, 2017, 2019, 2020)	13.92	1.41	198	March 2022	3.32	4/9
2	NZ → SWT Nov Y1	Non-proven	5.51	7.56	291	March 2022, February & April 2024	4.69, 4.15, 3.51	7/9, 7/9, 6/9
3	SWT (Y1), NZ (Y2)	Proven (2013, 2016, 2020)	7.39	0.56	29	March 2022	4.60	5/9
4	NZ	Non-proven	1.60	1.92	67	March 2022	3.90	5/9
5	PL	Proven (2013, 2019, 2021, 2022)	3.68	4.43	216	Not conducted		

**Table 2:** Summary statistics for linear mixed models predicting trends in Tt metabolite concentrations in male Owston’s civets. We present the degrees of freedom (d.f.), F- and *p*-values from ANOVA analyses, as well as the model AIC, conditional R^2^, random intercept variance (mean ± standard deviation) and adjusted intraclass correlation coefficient (ICC). We conducted Tukey post hoc contrasts only where variables were significant in LMMs and present the regression coefficient and standard error (β ± SE) as well as the *p*-value and 95% confidence intervals (CI). Models are ranked by AIC. Bold values indicate significant values; only significant contrasts are shown. Acronyms: Au = autumn, Wi = winter, Sp = spring, Su = summer.

Model	Factors	d.f.	F Value	*p* Value	AIC	R^2^_conditional_	Random Variance	ICC_adjusted_	Contrast	Β ± S.E.	*p* Value	95% CI
1	Season	1, 3	22.57	**<0.0001**	1823.89	0.15	0.23 ± 0.76	0.08	Sp-Au	0.41 ± 0.08	**<0.0001**	0.21, 0.62
									Wi-Au	0.52 ± 0.08	**<0.0001**	0.32, 0.72
									Su-Sp	−0.39 ± 0.08	**<0.0001**	−0.60, −0.18
									Wi-Su	0.50 ± 0.08	**<0.0001**	0.30, 0.70
2	Reproductive status	1, 1	7.60	0.07	1872.12	0.05	0.09 ± 0.80	0.01				
3	NULL	1, 773	18.00	**<0.0001**	1872.15	0.05	0.18 ± 0.80	0.05				
4	Age	1, 2	3.64	0.22	1874.75	0.05	0.10 ± 0.80	0.02				

**Table 3:** Reproductive organ measurements from male Owston’s civets. Note that semen parameters were calculated from pooled samples collected via urethral catheterisation and electroejaculation. The semen extender used for assessments was the Elite Kennel Fertility freezing medium (UK). Acronyms: BCS = body condition score, *p* = proven, NP = non-proven.

Male	Reproductive Status	Age (Years)	BCS	Reproductive Organ Measurements				Semen Parameters ^1^								
				Testis (L) Area (cm^2^)	Testis (R) Area (cm^2^)	Prostate Area (cm^2^)	Epididymis Width (mm)	Total Volume (µL)	Concentration (×10^6^/mL)	Motility (%)	Progressive Motility (%)	Progressive Motility (1–5)	Viability (%)	Normal Sperm (%)	Primary Defects (%)	Secondary Defects (%)
1	P	16.94	4/9	2.99	2.60	3.00	1.9	47	170	16	7	2	29	5	65	30
2	NP	8.87	7/9	3.04	2.85	3.60	2.2	23	294	15	13	1	31	7	38	55
3	P	10.78	5/9	3.15	3.08	2.40	2.7	35	0	-	-	-	-	-	-	-
4	NP	1.94	5/9	3.40	3.20	2.40	2.2	45	216	27	15	1	31	1	4	95

^1^ No sperm were found in the sample from male 3. This may be due to recent ejaculation and does not necessarily signify that this male is sterile.

**Table 4:** Repeat semen parameters from male 2. The semen extender used for assessments was Medium 199 (Germany). Acronyms: *p* = proven, NP = non-proven, UC = urethral catheterization, EE = electroejaculation. Please note that progressive motility was not assessed during these reproductive assessments and that viability and sperm morphology were assessed from pooled samples.

Male	Reproductive Status	Age (Years)	Method	Total Volume (µL)	Concentration (×10^6^/mL)	Motility	Viability (%)	Normal Sperm (%)	Primary Defects (%)	Secondary Defects (%)
2	NP	10.79	UC	20	2189.5	48	Not conducted	47	41	12
			EE	35	137.1	47				
		10.98	UC	70	714.95	20	72	16	33	51
			EE	215	31.23	60				

## Data Availability

The raw data supporting the conclusions of this article will be made available by the authors on request. Each institution owns their data and reserves the right to review who has access to the original unprocessed data.

## Supplementary Materials

The following supporting information can be downloaded at: https://www.mdpi.com/article/10.3390/ani16172677/s1: Table S1. Summary of anaesthetic protocols and drugs used during each assessment. Table S2. Body condition scoring criteria from Species360 [21]. Figure S1. Parallelism curves of serially diluted standards and pooled male samples. Serial dilutions of faecal extract yielded a displacement curve parallel to the standard curve (y = 1.054x − 2.02, R^2^ = 0.994, F_1,7_ = 1178.502, *p* < 0.0001). Figure S2. Weekly faecal testosterone metabolite concentrations across multiple years for males (A) 1, (B) 3, and (C) 4. Grey and black lines denote different collection years.

## Abbreviations

The following abbreviations are used in this manuscript:

Tt	Testosterone
ART	Assisted Reproductive Technology
IUCN	International Union for the Conservation of Nature
UK	United Kingdom
NZ	Newquay Zoo
PL	Port Lympne Wild Animal Park
SWT	Shaldon Wildlife Trust
TH	Thrigby Hall Wildlife Gardens
RT	Room temperature
CV	Coefficient of variation
BCS	Body condition score
UC	Urethral catheterization
EE	Electro-ejaculation
LMM	Linear mixed model
AIC	Akaike’s information criterion
PELT	Pruned exact linear time
CROPS	Changepoint for a range of penalties
GLS	Generalised least squares
P	Proven
NP	Non-proven
