# Supplementary material for: Reproductive Physiology and Reproductive Parameters of Male Owston’s Civets (Chrotogale owstoni)
Source: Animals (Basel). 2026 Aug 26;16(17):2677. doi: 10.3390/ani16172677 (PMC13564339; doi:10.3390/ani16172677)
Supplement: Supplementary file 1 [file animals-16-02677-s001.zip › animals-4504915-supplementary.pdf]

Reproductive physiology and reproductive parameters of male Owston's civets (*Chrotogale owstoni*)

Veronica B. Cowl *, Isabel Callealta, Christa S. van Wessem, Tullis Matson, Zak Showell, Susan L. Walker, Rebecca Mogey, Kathy Baker, Scott Bird, Jane Hopper and Katie L. Edwards

Supplementary Materials

Table S1. Summary of anaesthetic protocols and drugs used during each assessment.

Male	Induction	Maintenance	Reversal
1	Medetomidine 0.05mg/kg + Ketamine 2mg/kg IM	Sevoflurane	Atipamezole 0.25mg/kg
2	Chamber - sevoflurane	Isoflurane + Medetomidine 0.03mg/kg	Atipamezole 0.15mg/kg
3	Chamber - Sevoflurane	Sevoflurane + Medetomidine 0.03mg/kg	Atipamezole 0.15mg/kg
4	Medetomidine 0.05mg/kg + Ketamine 2mg/kg	Sevoflurane	Atipamezole 0.25mg/kg

Table S2. Body condition scoring criteria from Species360 [21].

Score	Body fat estimate	Criteria
1	Emaciated	Animal extremely emaciated; spinous processes, ribs, tail head, hip joints and lower pelvic bones projecting prominently; bone structure of shoulders and back easily noticeable; no fatty tissue can be felt.
2	Very underconditioned	Animal emaciated; slight fat covering over base of spinous processes; transverse processes of lumbar vertebrae feel rounded; spinous processes, ribs, tail head, hip joints and lower pelvic bones prominent; shoulders and back structure faintly discernible.
3	Moderately underconditioned	Fat buildup about halfway on spinous processes; transverse processes cannot be felt; slight fat cover over ribs; spinous processes and ribs easily discernible; tailhead prominent, but individual vertebrae cannot be identified visually; hip joints appear rounded but easily discernible; lower pelvic bones not distinguishable; shoulders and neck accentuated.
4	Slightly underconditioned	Slight ridge along back; faint outline of ribs discernible; tailhead prominence depends on conformation; fat can be felt around it; hip joints not discernible; shoulders and neck not obviously thin.
5	Ideal condition	Back is flat (no crease or ridge); ribs not visually distinguishable but easily felt; fat around tailhead beginning to feel spongy; shoulders and neck blend smoothly into body.
6	Slightly overconditioned	Slight crease down back; fat over ribs spongy; fat around tailhead soft; fat beginning to be deposited behind shoulders and along sides of neck.
7	Moderately overconditioned	Crease down back; individual ribs can be felt, but noticeable filling between ribs with fat; fat around tailhead soft; fat deposited along behind shoulders and along neck.
8	Very overconditioned	Crease down back; difficult to feel ribs; fat around tailhead very soft; area behind shoulder filled with fat; noticeable thickening of neck; fat deposited along inner thighs.

9	Obese	Obvious crease down back; patchy fat appearing over ribs; bulging fat around tailhead, behind shoulders and along neck; fat along inner thighs may rub together; flank filled with fat.

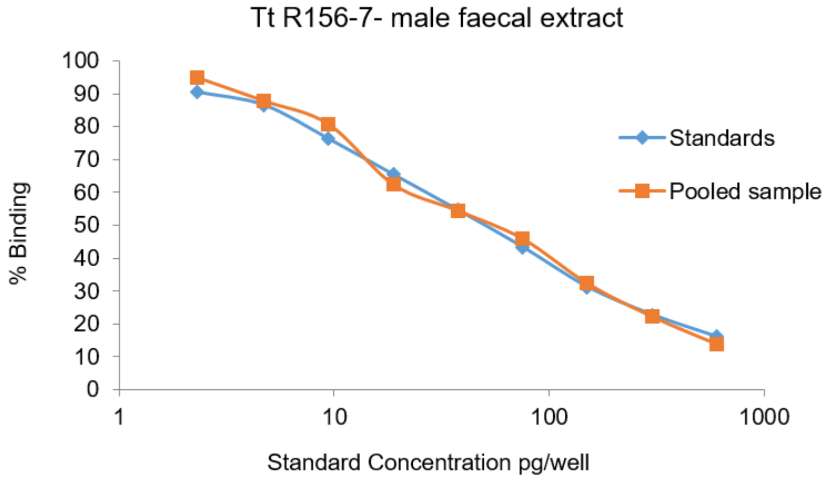

Figure S1. Parallelism curves of serially diluted standards and pooled male samples. Serial dilutions of faecal extract yielded a displacement curve parallel to the standard curve (y = 1.054x -2.02, R^2= 0.994, $F_{1,7}$= 1178.502, p <0.0001).

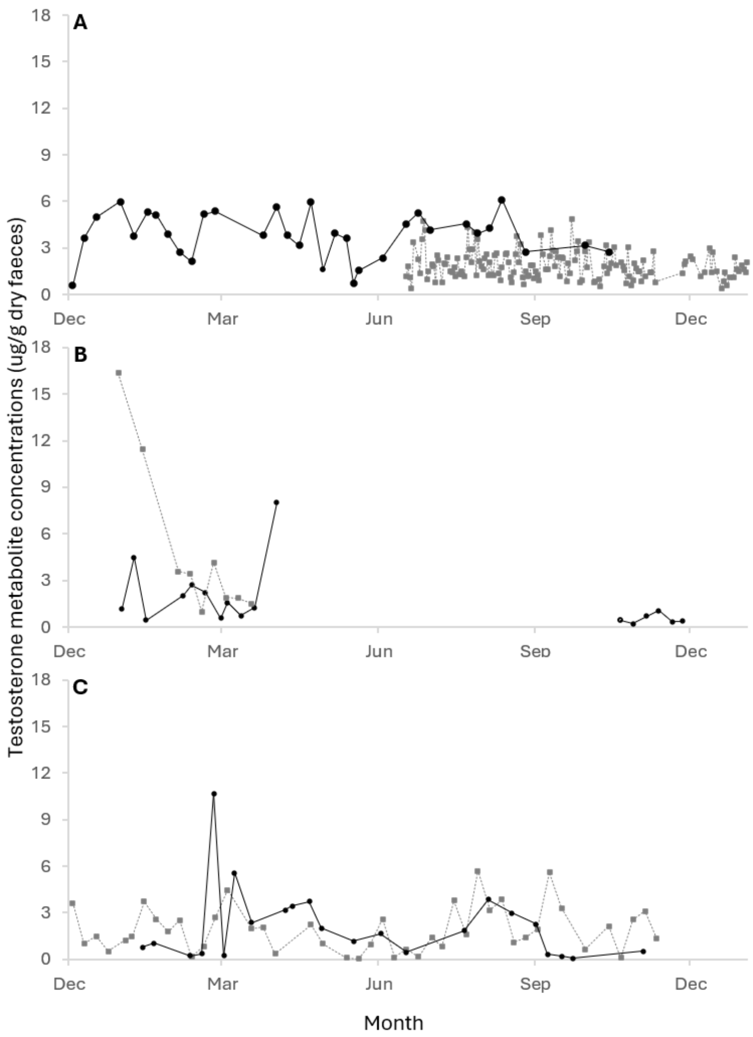

Figure S2. Weekly faecal testosterone metabolite concentrations across multiple years for males (A) 1, (B) 3, and (C) 4**.** Grey and black lines denote different collection years.
